# Premature Infants With Pulmonary Complications Exhibit Decreased Skin Maturity Early After Birth: A Cross‐Sectional Study

**DOI:** 10.1002/ppul.71562

**Published:** 2026-04-06

**Authors:** Samanta Cris Monteiro Frota, Luiza Eduarda Silva de Macedo, Larissa Queiroz Oliveira, Ingrid Fonseca Damasceno Bezerra, Anna Christina do Nascimento Granjeiro Barreto, Ingrid Guerra Azevedo, APPLE Consortium, Roberta Lins Gonçalves, Daniele Soares‐Marangoni, Gabriela Silveira Neves, Carolina Daniel de Lima‐Alvarez, Silvana Alves Pereira

**Affiliations:** ^1^ Januario Cicco Teaching Maternity, Brazilian Hospital Services Company (EBSERH) Federal University of Rio Grande do Norte Natal Brazil; ^2^ Vicerrectoría Académica ‐ Universidad Católica de Temuco, La Araucanía Temuco Chile; ^3^ Federal University of Amazonas and Federal University of Juiz de Fora Manaus and Juiz de Fora Brazil; ^4^ Federal University of Mato Grosso do Sul Campo Grande Brazil; ^5^ Hospital Sophia Feldman Belo Horizonte Brazil; ^6^ Graduate Program in Physical Therapy Federal University of Rio Grande do Norte Natal Brazil

**Keywords:** Neonatal Intensive Care, neonatal respiratory distress syndrome, preterm newborn, skin

## Abstract

**Introduction:**

Early identification of respiratory risk in preterm newborns (PTNB) remains challenging, as direct assessment of pulmonary maturity is often unavailable or requires invasive or resource‐intensive methods. Non‐invasive biomarkers capable of indicating physiological maturity shortly after birth could improve early clinical decision‐making in neonatal care. Because skin and lung development share common biological pathways, skin maturity assessed by optical reflectance may serve as an indirect marker of pulmonary development.

**Objective:**

This study evaluated the association between skin maturity estimated by skin reflectance and respiratory outcomes in PTNB during the first 48 hours of life.

**Methods:**

A cross‐sectional study was conducted with PTNB born admitted within 48?h. Exclusion criteria included cutaneous malformations, or conditions interfering with dermal assessment. Skin maturity was measured using the non‐invasive Preemie‐Test® device. Respiratory outcomes, such as chest radiographic abnormalities, pneumothorax, atelectasis, and pulmonary hemorrhage, were defined by clinical and radiological criteria.

**Results:**

A total of 82 PTNB were included, 54.9% male. Skin reflectance ranged from 0.9 to 2.1?nm. Lower skin maturity was significantly associated with radiographic abnormalities (*p* = 0.01), pneumothorax (*p* = 0.01), pulmonary hemorrhage (*p* = 0.01), atelectasis (*p* = 0.01), and need for invasive ventilation (*p* = 0.01).

**Conclusions:**

These findings suggest skin reflectance may serve as a rapid, objective, and non?invasive screening tool for neonatal respiratory risk.

## 1 Introduction

Prematurity, defined as birth before the completion of 37 weeks of gestation, represents a global public health challenge and remains one of the leading causes of neonatal morbidity and mortality, particularly in low‐ and middle‐income countries [1]. It is a multifaceted phenomenon influenced by biological, sociodemographic, environmental, and clinical determinants.

In Brazil, between 2012 and 2019, there was a slight but consistent reduction in the proportion of preterm births, from approximately 10.87%–9.95%, with the lowest rate recorded in 2015 (9.77%). However, despite this overall decline, women aged 45 years or older and those with fewer prenatal care visits had prematurity rates ranging from 14.88% to 17.92%, showing an upward trend over the study period. These findings underscore that, while overall indicators have improved, persistent inequalities demand targeted strategies for prevention and care [1].

Several complications are associated with preterm birth [2]. Among the most significant are respiratory disorders resulting from the structural and functional immaturity of the pulmonary system, often requiring ventilatory support in the first hours of life [3]. Such manifestations include findings such as atelectasis, nonspecific radiological abnormalities, pulmonary hemorrhage, and other clinical conditions that compromise the respiratory stability of preterm newborns (PTNB) [4, 5].

In this context, strategies for the early assessment of pulmonary maturity have been investigated to support clinical decision‐making and minimize the risk of complications. Among the proposed methods, non‐invasive skin evaluation has emerged as a potential marker of fetal maturity. This is based on the fact that the epidermis and the nervous system share the same embryonic origin (ectoderm), while the lung, although derived from the endoderm, develops through molecular signaling pathways common to both, which may explain the observed correlation between skin and lung maturity [6, 7].

The process of skin keratinization, culminating in the formation of the functional stratum corneum, occurs progressively and becomes more complete around the 34th week of gestation. In extremely preterm newborns, this layer is immature and thin, compromising the skin's barrier function and increasing the risk of heat loss, dehydration, infections, and, indirectly, respiratory complications [8]. Emerging evidence suggests a possible correlation between skin and lung maturity, which supports the interest in using dermal parameters as indirect indicators of respiratory development in PTNB [8, 9].

In this regard, optical technologies that analyze skin reflectance based on the interaction of light with the skin have proven feasible for estimating gestational age and potentially organ maturity [10, 11]. Non‐invasive methods based on light scattering, using photodetectors and specific wavelengths, can measure epidermal thickness, cell density, and the degree of keratinization [12]. Multicenter studies conducted with more than 700 newborns have demonstrated high accuracy of these tools in estimating gestational age, suggesting their potential as biomarkers of neonatal physiological maturation [13, 14]. Therefore, the present study aimed to evaluate the relationship between skin maturity estimated by skin reflectance, and respiratory outcomes in PTNBs within the first 48 h of life.

## 2 Methods

### 2.1 Study Setting and Period

This was a cross‐sectional observational study, derived from a larger prospective cohort registered on the REBEC platform (RBR‐10ch623r; UTN U1111‐1302‐9740) and conducted in accordance with the STROBE checklist guidelines (Malta et al., 2010) [15]. The study was carried out between March 2024 and February 2025 in the Neonatal Intensive Care Unit (NICU) of Maternidade Escola Januário Cicco, located in Natal, Brazil. The maternity hospital provides care exclusively to patients of the Brazilian Unified Health System, serves as a state reference center for high‐risk deliveries, has 20 NICU beds, and admits between 150 and 180 preterm newborns (PTNBs) annually (local data, 2023). The study was approved by the Research Ethics Committee (Approval No. 6.523.532), in compliance with CNS Resolution 466/2012.

### 2.2 Participants and Eligibility Criteria

The study population consisted of PTNB with a gestational age of < 37 weeks, admitted to the NICU within the first 48 h of life, whose parents or legal guardians signed the informed consent form. PTNB with cutaneous malformations, congenital heart disease, confirmed infections, or other conditions that could interfere with skin assessment (e.g., anhydramnios, hydrops, congenital skin diseases, or chorioamnionitis) were excluded, as well as newborns with patent ductus arteriosus, tachypnea not attributable to prematurity, or clinical or laboratory‐confirmed infections.

Sample size was calculated using G*Power 3.1, considering a two‐tailed Student's test for independent samples. The calculation was based on preliminary data on skin reflectance in PTNBs with and without respiratory distress syndrome [8]. A Cohen's d effect size of 0.9, statistical power (1–β) of 0.95, significance level (α) of 0.05, and an allocation ratio of 1:1 were adopted. Based on these parameters, the required sample size was 68 participants. To compensate for potential losses or exclusions, the sample was increased by 20%, totaling 82 PTNB.

### 2.3 Procedures

#### 2.3.1 Participant Characterization

Data were collected through medical record review and included maternal variables (age, number of prenatal visits, mode of delivery, antenatal corticosteroid use) and neonatal variables (gestational age, sex, birth weight, and Apgar scores at 1 and 5 min).

Respiratory outcomes were diagnosed based on clinical and radiographic criteria. Atelectasis was considered when chest X‐ray showed homogeneous or heterogeneous opacities associated with pulmonary volume loss, fissure or mediastinal shift, and diaphragmatic elevation. Pneumothorax was defined as the presence of a visceral pleural line outlining a collapsed lung, associated with peripheral hyperlucency without visible vascularization; in tension pneumothorax, mediastinal shift and diaphragmatic flattening were also observed. Pulmonary hemorrhage was defined as the presence of blood during tracheal aspiration associated with diffuse and bilateral alveolar opacities, often in a “butterfly‐wing” pattern, or with asymmetric consolidations, possibly accompanied by air bronchograms. Chest radiographic abnormalities were recorded when there was an increase in perivascular markings not classifiable under the aforementioned categories [8, 13, 14].

**FIGURE 1:**
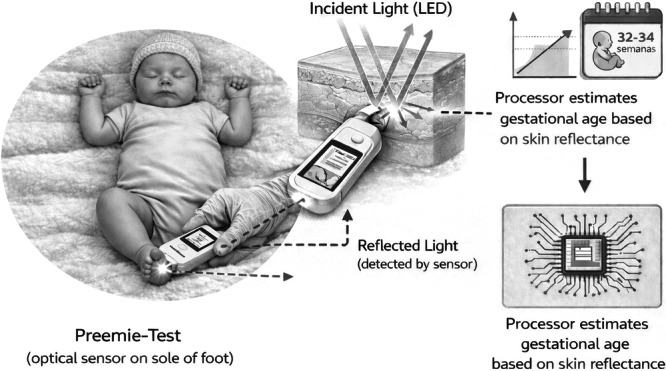
Skin maturity assessment using the Preemie‐Test (PreemieTech®, model PT‐1000). The optical sensor is applied to the plantar surface of the newborn's heel with the infant in the supine position. The device emits light (400–1200 nm) and detects the light reflected by the skin; a processor converts the reflectance signal into an estimate of gestational age/skin maturity. Generative AI was used to generate the initial concept image for Figure 1; the authors reviewed, edited, and took responsibility for the final figure.

Skin maturity was assessed using the Preemie‐Test device (PreemieTech®, model PT‐1000), a non‐invasive optoelectronic instrument. The device estimated skin maturity through reflectance, emitting light between 400 and 1200 nm and performing 30 automatic measurements (3 touches of 5 s each). All measurements were performed by four trained evaluators, following the standardized collection protocol (Figure 1).

#### 2.3.2 Collection Protocol

Measurements were taken on the plantar surface of the heel, with the PTNB in the supine position. The examiner placed the device in contact with the newborn's skin three times at the same site, for 5 s each, as indicated by the device's display. Once the sensor touched the skin, the device automatically triggered 10 measurements per touch, with the final result calculated as the median of 30 values. The emitted light interacted with the skin, being scattered, and the reflected light was captured by the sensor, processed by a control unit, and stored for analysis. Error alerts (e.g., ambient light entering the sensor) were generated in cases of involuntary movement of the newborn or examiner, requiring a new attempt (Reis et al., 2022) [10]. The device was reusable and disinfected with 70% alcohol before and after each use, and calibration was performed at the beginning and end of the study.

### 2.4 Statistical Analysis

Data normality was assessed using the Kolmogorov‐Smirnov test. Quantitative variables were expressed as mean ± standard deviation, and qualitative data were presented as frequency and percentage. The independent‐samples Student's *t* test was used to compare mean skin reflectance between groups with and without respiratory complications (invasive mechanical ventilation, atelectasis, radiographic abnormalities, pulmonary hemorrhage, and pneumothorax). A significance level of 5% was adopted. All analyses were performed using the Statistical Package for the Social Sciences (SPSS), version 25 (IBM Corp., USA).

## 3 Results

**FIGURE 2:**
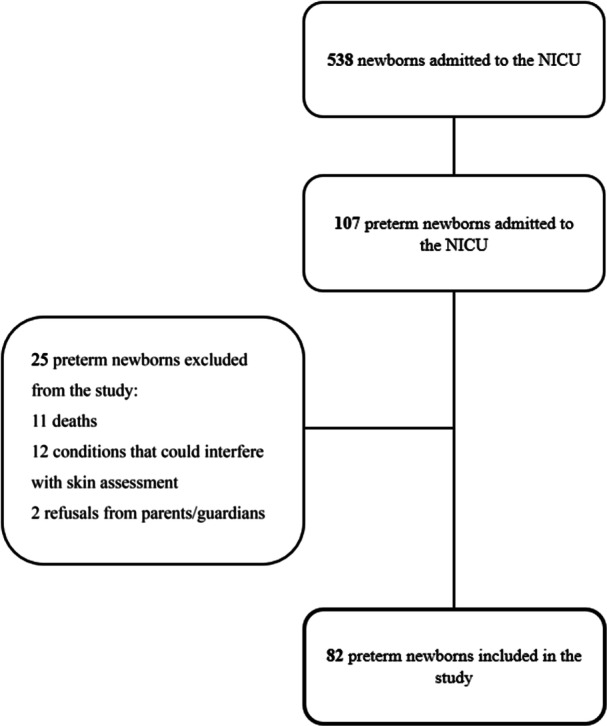
Flowchart. NICU, Neonatal Intensive Care Unit.

During the study period, the NICU admitted 538 newborns, of whom 107 were preterm. Among these, 82 PTNB met the eligibility criteria and were included in the study. Figure 2 presents the sample flowchart.

Of the 82 PTNB evaluated, 54.9% (*n* = 45) were male, and 61% (*n* = 50) were delivered by cesarean section. Table 1 presents the descriptive characteristics of the sample. Skin reflectance, the variable representing skin maturity, ranged from 0.9 to 2.1 nm.

**TABLE 1:** Maternal and neonatal characteristics (*n* = 82).

Variable	Mean ± SD/Frequency (%)
**Maternal variables**	
Age (years)	29 ± 7.8
Number of prenatal visits	7 ± 2.7
Antenatal corticosteroid use	
Yes	50 (61%)
No	32 (39%)
**Neonatal variables**	
Gestational age (weeks)	33 ± 2.3
Birth weight (g)	1913.6 ± 517.0
Apgar score at 1 min	7 ± 2
Apgar score at 5 min	8 ± 1
Head circumference (cm)	31 ± 2.2
Skin maturity (nm)	1.6 ± 0.2

Abbreviations: cm, centimeters; nm, nanometers; g, grams; SD, standard deviation.

Comparative analysis showed that PTNB with chest radiographic abnormalities had lower skin maturity than those without abnormalities (*p* < 0.01). Similarly, lower values of skin maturity were associated with the presence of pneumothorax (*p* < 0.01), atelectasis (*p* < 0.01), pulmonary hemorrhage (*p* < 0.01), and the use of invasive mechanical ventilation (*p* < 0.01). Table 2 presents skin maturity values across all respiratory outcomes analyzed.

**TABLE 2:** Respiratory outcomes and skin reflectance (*n* = 82).

Respiratory Outcome	*n*	Skin reflectance (nm) Mean ± SD	*p*‐value
**Chest radiographic abnormality**			
Yes	24	1.49 ± 0.19	*p* < 0.01
No	58	1.62 ± 0.22	
**Pneumothorax**			
Yes	4	1.33 ± 0.23	*p* < 0.01
No	78	1.60 ± 0.21	
**Atelectasis**			
Yes	15	1.45 ± 0.21	*p* < 0.01
No	67	1.62 ± 0.20	
**Pulmonary hemorrhage**			
Yes	2	1.09 ± 0.14	*p* < 0.01
No	80	1.59 ± 0.20	
**Invasive mechanical ventilation**			
Yes	25	1.48 ± 0.17	*p* < 0.01
No	57	1.62 ± 0.21	

Abbreviations: nm, nanometers; SD, standard deviation.

## 4 Discussion

The objective of this study was to evaluate skin reflectance in PTNB during the first 48 h of life and its association with respiratory outcomes. The findings indicate a relationship between lower skin maturity and the presence of respiratory complications in PTNB. Preterm infants with chest radiographic abnormalities, those requiring invasive mechanical ventilation, and those with pulmonary hemorrhage, pneumothorax, or atelectasis demonstrated lower indices of skin maturity within the first 48 h of life. These results suggest a possible relation between skin immaturity, as measured by skin reflectance, and insufficient pulmonary development.

A study conducted by Neves et al. (2024) [8] supports our findings by demonstrating an association between skin immaturity and the occurrence of respiratory distress syndrome (RDS). Although the present study did not specifically analyze RDS, the respiratory outcomes evaluated, particularly radiographic abnormalities, atelectasis, and mechanical ventilation, are clinical manifestations frequently associated with this condition.

In two multicenter clinical studies conducted in Brazil [10] and Mozambique [16], the same research group (Reis et al.) [10, 16] reported that skin reflectance, when combined with birth weight and antenatal corticosteroid exposure, predicted RDS with an overall accuracy of 89.7% [10]. These results corroborate the present study, indicating that skin reflectance in newborns represents a robust marker not only for RDS but also for other respiratory outcomes.

One hypothesis to explain this finding is that different organ systems share similar developmental trajectories during fetal life, allowing features observed in one system, such as the skin, to serve as indirect indicators of the maturation of another, such as the lungs [12]. Experimental evidence from animal models has suggested that both the stratum corneum and pulmonary surfactant share common mechanisms related to lipid production, reinforcing the interdependence of skin and lung maturation processes [17].

In our sample, newborns with pulmonary hemorrhage showed lower skin reflectance values. This pattern reinforces the hypothesis that dermal parameters obtained by non‐invasive methods may contribute to the early stratification of respiratory risk, particularly in clinical contexts where direct assessment of pulmonary function is limited. In neonatal units with scarce resources, early identification of the risk for respiratory comorbidities is a crucial step for decision‐making and therapeutic planning. The possibility of using dermal parameters as indirect markers of lung maturity may broaden neonatal screening strategies, enabling earlier, individualized, and potentially more effective interventions [14, 18].

Several researchers have explored the use of portable and non‐invasive technologies for early diagnosis and monitoring in neonatology, expanding clinical assessment possibilities beyond skin analysis [19, 20]. For example, researchers in Canada have applied near‐infrared spectroscopy to monitor cerebral oxygenation in preterm infants, allowing early detection of neurological injury [21, 22]. Technologies for continuous monitoring of temperature and energy metabolism have also been applied in neonatal units, including low‐resource settings, demonstrating the feasibility of such approaches to improve neonatal care [23, 24]. These advances highlight the potential of portable and non‐invasive tools to enhance risk stratification and personalize interventions in newborns, while also minimizing handling, stress, and pain, factors that contribute to improved neurodevelopment.

The Preemie‐Test has been applied in low‐ and middle‐complexity contexts, particularly in regions with limited access to prenatal imaging or gestational follow‐up [15]. The device offers a viable alternative for estimating gestational age in delivery rooms, NICUs, medium‐sized maternity hospitals, pre‐hospital emergency care services, and even in home births with professional assistance. Its use is especially relevant in cases where early ultrasound is unavailable, gestational dating is uncertain, or rapid clinical decision‐making is required [10, 16].

Multicenter studies have investigated its applicability in diverse scenarios. In Brazil, it has been implemented in public hospitals across five states; in Mozambique, it has been tested in highly vulnerable contexts [15]; and in Portugal, it is being evaluated as a complementary tool to routine clinical practice. Beyond gestational age estimation, the device has also been explored as a tool to predict the risk of RDS, further expanding its potential role in neonatal risk stratification across different healthcare settings [6].

Despite its promising results, this study has some limitations. Conducted in a single hospital with a specific group of preterm infants, the findings may not fully reflect what occurs in other maternity settings. Furthermore, as certain newborns with specific conditions were excluded, the sample may not represent the full diversity of preterm infants. Another important consideration is that, although a validated device was used, factors such as the NICU environment or sensor application technique may influence measurements. Future studies with larger samples and conducted in multiple settings may help confirm and expand upon these results.

## Author Contributions

**Samanta Cris Monteiro Frota, Luiza Eduarda Silva de Macedo, and Larissa Queiroz Oliveira:** conceptualization, investigation, data curation, and writing – original draft preparation. **Ingrid Fonseca Damasceno Bezerra and Anna Christina do Nascimento Granjeiro Barreto:** investigation, data curation and writing – review and editing. **Ingrid Guerra Azevedo:** formal analysis, methodology, and writing – review and editing. **APPLE Consortium:** contributed to multicenter study design, data collection infrastructure, and collaborative scientific discussions. **Roberta Lins Gonçalves, Daniele Soares‐Marangoni, and Gabriela Silveira Neves:** methodology, supervision, and writing – review and editing. **Carolina Daniel de Lima‐Alvarez:** conceptualization, methodology, supervision, and writing – review and editing. **Silvana Alves Pereira:** conceptualization, methodology, supervision, project administration, funding acquisition, and writing – review and editing.

## Conflicts of Interest

The equipment used in the study was provided by Birthtech® Dispositivos para a Saúde Ltda., and funding for its acquisition was partially supported by Grand Challenges Canada [R‐TTS‐2309‐60401], which supports the Bold Ideas with Great Impact® program. Neither entity participated in the study design, data collection, analysis, or interpretation.

## Supporting information

suplemental_material.

## Data Availability

The data that support the findings of this study are available from the corresponding author upon reasonable request.
