# Supplementary material for: Premature Infants With Pulmonary Complications Exhibit Decreased Skin Maturity Early After Birth: A Cross‐Sectional Study
Source: Pediatr Pulmonol. 2026 Apr 6;61(4):e71562. doi: 10.1002/ppul.71562 (PMC13051638; doi:10.1002/ppul.71562)
Supplement: Supplementary file 1 — suplemental_material. [file PPUL-61-0-s001.docx]

**Authors:**

1. **Silvana Alves Pereira**^5^ - ORCID: 0000-0002-6226-2837) - silvana.alves@ufrn.br
2. **Roberta Lins Gonçalves**^1,6^ – ORCID: 0000-0003-4976-0716 – betalinsfisio@ufam.edu.br
3. **Daniele Soares-Marangoni**^2^ - ORCID: 0000-0002-5354-5456 - daniele.soares@ufms.br
4. **Lysien I. Zambrano**^3^ **-** ORCID: [0000-0001-9002-5807](https://orcid.org/0000-0001-9002-5807) **-** lysien.zambrano@unah.edu.hn
5. **Gabriela Silveira Neves**^4^ **–** ORCID: 0000-0001-6765-2968 - neves.gabriela87@gmail.com
6. **Alexandre Lopes Miralha**^1^ **-** ORCID: 0000-0001-8104-7086- alexmiralha@ufam.edu.br
7. **Sinval Sousa da Costa Neto**^1^ - ORCID: 0009-0009-0126-946X - sinval92@gmail.com
8. **Abel Valente da Silva**^1^ **-** ORCID: 0009-0007-7364-9416 - abelsmg@hotmail.com
9. **Tainá Costa Pereira Lopes**^1^ **-** ORCID: 0000-0002-2867-7027 **-** Tainapereiralopes@outlook.com
10. **Julie Andrez Ramos de Andrade Vieira**^1^ **-** ORCID: 0009-0008-9097-0970 - julie.vieira@ufam.edu.br
11. **Amanda Valente da Silva**^1^ **-** ORCID: 0009-0001-2507-9080 - amanda.valente@ufam.edu.br
12. **Ana Júlia Teixeira Costa**^1^ **-** ORCID: 0009-0007-2219-7472 - costa.ana@ufam.edu.br
13. **Evelyn Cristinne Costa Lima**^1^ **-** ORCID: 0009-0009-0262-3432 - evy.c.clima@gmail.com
14. **Nayara Bezerra Bessa**^1^ **-** ORCID: 0009-0004-1162-3913 – nayarabbessa3@gmail.com
15. **Barbara Lira Bahia**^1^ **-** ORCID: 0000-0002-8363-3211 **-** fisio.barbara@hotmail.com
16. **Pérsida Gomes de Souza Rocha**^5^ **-** ORCID: 0009-0002-0385-168X) -[persida.gsouza@gmail.com](mailto:persida.gsouza@gmail.com)
17. **Francelio Silva de Sousa Júnior**^5^ - ORCID: 0009-0000-6469-0643) -franceliojunior@gmail.com
18. **Júlio Cesar Bertoso Lima**^2^ **-** ORCID: 0009-0008-4488-0712) - jbertosodelima@yahoo.com.br
19. **Hanna Letticia Oliveira Lima**^5^ **-** ORCID: 0009-0008-7073-6212) -hanna.oliveira.016@ufrn.edu.br
20. **Roseany Cavalcante da Silva**^5^ **-** ORCID: 0009-0004-3012-3228) - roseanycavalcante@hotmail.com
21. **Giovanna Dantas de Araújo**^5^ **-** ORCID: 0009-0000-0403-8282) -giovanna.araujo.082@ufrn.edu.br
22. **Ariany Estefany da Silva**^5^ **-** ORCID: 0009-0002-7983-0420) -ariany.silva.130@ufrn.edu.br
23. **Ana Carolina Aguirres Braga**^2^ **-** ORCID: 0000-0002-2407-1642) - [sr.aguirres@gmail.com](mailto:sr.aguirres@gmail.com)
24. **Amanda de Oliveira Arguelho**^2^ **-** ORCID: 0009-0007-4075-9256) -amanda.arguelho@ufms.br
25. **Geovana Beatriz Pereira Mendes**^2^ **-** ORCID: 0009-0008-7927-3736) [-geovana.beatriz@ufms.br](mailto:-geovana.beatriz@ufms.br)
26. **Ellen Kathellen Sá de Souza^1^**  -ORCID: 0000-0001-8428-7683 - ellen.kathellen@hotmail.com
27. **Iris Vicuña Julião Dias**^2^ **-** ORCID**:**  0009-0002-9271-8732 - iris.dias@ufms.br
28. **Mariana Tavares Martines**^2^ **-** ORCID: 0009-0002-9505-8186) - mariana.tavares.m@ufms.br
29. **Maria Luiza Kohatsu Arakaki**^2^ **-** ORCID: 0009-0000-5429-4794) - maria_arakaki@ufms.br
30. **Géssy Cardoso Santos**^2^ **-** ORCID: 0009-0003-9971-7617 [-gessy.cardoso@ufms.br](mailto:-gessy.cardoso@ufms.br)
31. **Talita Cândida da Silva Almas Cabral**^6^ - ORCID: 0009-0007-7634-9013 - talitacandidajf@gmail.com
32. **Gessilene Buzaglo Mori**^7^ – ORCID: 0009-0005-1348-3309 -Gessilenebuzaglo9@gmail.com
33. **Janderson Nascimento**^7^ **-** ORCID: 0009-0000-2943-8194 - [janderson.buzaglo71@gmail.com](mailto:janderson.buzaglo71@gmail.com)
34. **Itzel Carolina Fuentes Barahona**^3^ – ORCID: [0000-0002-9995-2276](https://orcid.org/0000-0002-9995-2276) - itzel.fuentes@unah.edu.hn
35. **Ana Michell Lopez**^3^ – ORCID: 0009-0001-2306-163 - alopez@unah.edu.hn
36. **Ivan Espinoza**^3^: ORCID: 0000-0003-4144-7135 - ivan.espinoza@unah.edu.hn
37. **Eleonora Espinoza**^3^: ORCID: 0000-0001-7218-3481- eleonora.espinoza@unah.edu.hn
38. **Henry Castro**^3^: ORCID: 0000-0002-7679-6602 - henry.castro@unah.edu.hn
39. **Héctor Manuel Carranza Coello**^3^: ORCID: 0009-0001-2306-1637 - carranzacoello@yahoo.com
40. **Amanda Resende Chagas**^4^ **-** ORCID: 0009-0006-1744-7142 **–** [resende.amanda.c@gmail.com](mailto:resende.amanda.c@gmail.com)
41. **Scheila Tompai Marinho**^4^ **-** ORCID: 0009-0008-9296-1381 – scgetm@gmail.com
